# Molecules Editorial: 30 Years of Molecules—An Editor’s Experiences and Visions for the Future

**DOI:** 10.3390/molecules31050905

**Published:** 2026-03-09

**Authors:** Thomas J. Schmidt

**Affiliations:** University of Münster, Institute of Pharmaceutical Biology and Phytochemistry, PharmaCampus-Corrensstrasse 48, D-48149 Münster, Germany; eicmol@uni-muenster.de

Dear readers, authors, reviewers, editors, coworkers and staff of *Molecules*!

It is my great pleasure to cordially greet you all on the occasion of our journal’s 30th anniversary. Indeed, 30 years ago, on 21 March 1996, the inaugural issue of *Molecules* was published, then containing only a single article (Contribution 1). But the journal grew quickly, and since then, over 60,000 articles have been published, making *Molecules* one of the largest online journals in chemistry. In fact, *Molecules* now covers the full breadth of chemistry, comprising 25 sections across all of chemistry’s diverse disciplines, guided by an academic editorial board of >1700 renowned international experts and publishing about 5000 papers per year. All of this would not be possible without the diligent and dedicated work of the staff working in the editorial office branches across the globe, an army of voluntary expert reviewers and guest editors and, of course, thousands upon thousands of authors from all branches of chemistry. In 1996, with the Internet still in its early years, it would almost certainly not have been possible to foresee that *Molecules* would exhibit such sharp growth and become as successful as it has in the years since (for details on our history, see Contributions 2 and 3), but clearly the founder of *Molecules* and the publishing company MDPI, as well as *Molecules’* first author (Contribution 1) and first Editor-in-Chief, Shu-Kun Lin, was on the right track. Now, 30 years later, MDPI is the home of over 500 diverse open access journals across all fields of science, engineering and humanities, where 4.5 million authors have published their work over the years; it is often forgotten that our journal, *Molecules*, was the very starting point of it all and, in fact, one of the very first full open access science journals ever. Even though early roots of an “open access” (OA) movement can be traced back to the 1950s, its full potential was only realized with the advent of the internet and, thus, the timely creation of *Molecules* can be considered a truly visionary act in the history of chemistry and scientific publishing. 

It is my personal honor to serve as the journal’s Editor-in-Chief (EiC) at this very moment, and it is a privilege to have the opportunity to share a few thoughts on my past experiences with *Molecules* and my future visions for the journal in this Editorial. For me, the year 2026 also marks the 10th anniversary of my commitment as a Chief Editor of *Molecules,* since I became the section EiC for Natural Products Chemistry in 2016. Before that, I had already served as Guest Editor and Associate Editor for some years. I have thus had the pleasure of being involved in editorial work for *Molecules* for over a third of its life. As I have stated various times before (e.g., Contribution 4), it has overall been a very pleasant experience from my very first publication in *Molecules* to the present. I have encountered and interacted with very many wonderful coworkers at the editorial offices and other staff, so many great colleagues among the academic editors and reviewers as well as authors, that I consider it a great reward to be in this position. It is also rewarding to see the continuous outcome of this work in the form of so many excellent published examples of cutting-edge chemistry, and—from time to time coming directly to my address—positive feedback from authors, editors or reviewers. These are just a few of my personal pleasant experiences with the journal. Taken together, all this continues to reinforce my very positive feelings about our journal. Then, of course, I have the pleasure of following the year-by-year development of the numerical indicators, e.g., bibliometric data, reflecting the journal’s *success*, and ultimately its high *quality*. I can proudly say that the journal impact factor since 2024 has been on the rise again and is now back at 4.6 (a new JIF is expected this year). Even more excitingly, the CiteScore index has steadily risen from 5.9 in 2021 to 8.6 at present! Of course, these are just numbers, but they do reflect success and quality.

Naturally, the journal’s success is a direct consequence of its quality. This comprises all aspects of quality, from the authors’ experiences with the technical platform for submission via the reviewing and editorial decision process, to the quality of scientific content in the published work. All of this ultimately determines success in the form of views (almost 8.5 M in 2025), downloads (almost 1.7 M in 2025) and citations (about 280 K in 2025). Quality and success can be considered a function of the sum of pleasant experiences of thousands of authors and readers. At the same time, pleasant experiences and quality directly affect the journal’s *reputation*. Quality, in turn, is also a function of reputation. The better the reputation, the higher the possibility that leading scientists submit their excellent work. Thus, the journal’s quality, success and reputation mutually reinforce each other, forming a self-amplifying loop which I would like to call the “loop of success” (LOS; see Scheme 1).

Unfortunately, on the other hand, there are also antagonistic factors, obstacles that can obstruct the LOS. Not everything is always positive, not all experiences pleasant. There are also downsides.

The first one to come to my mind is the (not infrequent) question from “skeptical” colleagues about *Molecules* and MDPI journals being “predatory”. It is a well-known fact that the reputation of Molecules and some other “gold” OA journals are under constant attack by self-declared advocates (whoever they are and whoever keeps them going) of “honest publishing” who continuously repeat the long-disproven narrative of Molecules as a “predatory journal”. Such rumors are very easily spread, seemingly at the speed of light these days. They are completely disregarding the full compliance of MDPI with COPE guidelines and the many measures taken by the journal and publisher to ensure and support publishing ethics in favor of fully transparent, open science. When asked such questions, I usually refer to the journal’s and publisher’s websites, where our principles concerning publication ethics and related aspects are openly accessible. And people are often surprised to find all this as public information if they follow my advice to read it. But of course, public statements must also be backed up accordingly by appropriate action. It is almost impossible to change people’s minds just with words once they have been convinced by false rumors. The only way to counteract such rumors and slander is to disprove them by action and facts. And these must be provided with maximum transparency! Attacks out of the dark are easy in the environment where OA takes place. Openness, brightness and maximum transparency are the only ways to react to such attacks.

One important obstacle to our above-mentioned LOS is, in fact, the very environment in which OA scientific publishing must happen. And this is an obstacle that was not known in “classical” scientific publishing of previous times. The environment in which the more traditional science journals were (and still are) published—let me call it the “publishotope”—is a rather exclusive landscape that might be compared to a well-tended nature reserve with special protection, inhabited only (or mainly) by well-behaved truth-loving herbivores. While this reserve is restricted to the sheltered libraries of science institutions with access limited only to insiders, OA publishing must take place in a vast huge jungle with conditions changing quickly in a near-chaotic, lawless manner, co-inhabited by lots of evil wild beasts (the true predators) that experience little or no restrictions at all and do not care about truth and facts: the internet. Most of the huge flood of information in this space is not easily (if at all) verifiable, unreliable in many cases, with truthfulness always in question. False information not only spreads but is also multiplied at maximum speed. Tendencies to deliberately create and spread fake news, pseudo- and anti-science, to destabilize human society are on the rise and now ongoing in a practically unconcealed manner. Naturally, in *this* environment it is extremely demanding to publish true science seriously, based on honest investigation and sound data, and to keep it from being corrupted, because it must coexist alongside a vast, vast amount of bogus, lies and fake facts. In this publishotope, it is particularly important (and particularly difficult) to keep the published content “clean”, i.e., integer and sound!

As a consequence, the role of the EiC is sometimes, inevitably, accompanied by the not-so-pleasant experience of having to deal with examples of sloppiness, misconduct and sometimes even outright fraud, in review reports or in articles that require corrections or even retractions. Even though in the EiC’s perception of these cases may appear numerous (because he naturally sees most of them), they are, in fact, only a tiny, tiny fraction. In the preparation of this editorial, I convinced myself of this relieving fact and I thank the journal relations team for the numbers: In a three-year period (2023–2025), 163 ethics cases were closed in Molecules. The most frequent type of ethics issue by far was image manipulation, with 60 cases in total. The next most commonly occurring ethics concern was plagiarism (11 cases). As an outcome of these cases, there were 51 “Correction” decisions and 24 “Retraction” decisions. In relation to 18,145 (!) Articles and Reviews published in the same time span, these figures are almost negligible (about 0.13% retractions and 0.28% corrections, not counting necessary corrections asked for by authors). So let me be clear: The quality of the overwhelming vast majority of our publications is just excellent! Nevertheless, it must be an editor’s duty to search for possibilities to further minimize the small fraction of poor-quality work, in order to further decrease the fraction of unpleasant experiences in relation to pleasant ones and thus to remove obstacles from the LOS.

Let me, therefore, now come to some of my *visions* for the journal’s future, because visions are dreams of changing things for the better, so they must represent consequences of the less pleasant experiences.

Of course, it should be an essential goal to avoid the possibility that poor quality work gets published in the first place. And therefore, naturally, good peer review is key to this goal. The journal has done a lot in the past few years to improve the peer review process and to avoid poor-quality peer review. And new moves in this direction, such as an improved mechanism to select reviewers during the pre-review check, or the new “Co-Review” program where early-career scientists can perform reviews under the guidance of experienced Editorial Board Members as mentors, are currently under development. For me, one of the best measures taken so far in this respect is the introduction of *open peer review* (OPR). As an author, I have chosen this option ever since it was introduced, because I think that the course of the peer review process is an integral, essential part of each manuscript and almost as important as the final content itself. Certainly, most readers do not look up the publication’s history, but it may become important, e.g., during later discussions, how results were originally presented and what the reviewers’ part in the final version was. However, most articles published under the OPR scheme currently feature a semi-open form, i.e., the authors allow that the reviewers’ comments and authors’ replies are published with the papers, but the names of the reviewers remain secret. The reviewers can choose to sign their reports, but most of the time they do not use this option. I would like to advocate here for a *FULL* open peer review (FOPR), in which both the comments as well as the reviewers’ identity are made public. I would find this very desirable for various reasons, of which I will mention here only the most important one: If reviewers were aware that their names would be published, they would obviously take maximum care to write an excellent, balanced, detailed and well-argued review report. In the present “real world” of blind review, unfortunately, this is not always warranted. Also, I always found it a little strange that reviewers know who the authors are, but not vice versa. This is not the most proper setting for a fair dispute, if dispute is necessary. But of course, it will not be possible to make FOPR fully and generally mandatory “just like that”, because it might then become difficult to recruit reviewers. Of course, it is also understandable that reviewers are reluctant to reveal their identity in certain cases, where they may feel uneasy, e.g., to criticize, or even suggest rejection, of their peers’ work. But this attitude is due to the misconception of many (authors and reviewers), that review is somehow meant as an obstacle to publish rather than a means to improve the quality. However, I can foresee that FOPR, if scientists could be honestly convinced that this should be the normal way, would lead to a significant improvement in the quality of peer review and thus further improve the publications’ quality. I will therefore soon edit a pilot Special Issue myself (topic to be announced), in which the FOPR model will be the “new normal”. (Of course, contributions will be supported by some incentives for the reviewers and authors to participate.) If reviewers and authors were to gradually get used to FOPR as the new normal, I am convinced that this would lead to a dramatic rise in review and publication quality. I am sure many of the above-mentioned cases, where poor quality is detected in already published work, could be avoided. 

Besides peer review, I would endorse full openness in yet another aspect to improve quality. As mentioned above, the most frequent reason for ethics investigations is (alleged) image misuse. So, this is another significant obstacle to our mentioned LOS. Usually, during the investigations of such cases, the authors are asked to provide the original, uncropped and unaltered image material that was used in the production of figures under debate. And, unfortunately, very frequently, the authors do not find it possible to provide such material, which then—in severe cases—may lead to a loss of confidence in the integrity of a publication so that such work may be ultimately retracted. I would therefore also suggest requiring raw image data (and other raw data, such as spectroscopic/spectrometric or chromatographic) uploaded along with manuscripts when they are first submitted. Such full open raw data (FORD) submissions would then make it possible to store data immediately and permanently together with the publication itself and thus make them an integral part of the latter. I am sure this would avoid many of the cases where data integrity is later questioned. Of course, *Molecules,* as per its instructions for authors, requires that all raw data underlying a publication are stored by the authors for at least five years. However, in my opinion, this alone is not sufficient (and not timely) anymore. The data underlying published results should be stored as long as the publication itself, i.e., (hopefully) forever, and they should be stored where they are easiest to find: directly alongside the publication. Many authors (including myself) already provide curated data collections in the form of supplementary files. It would be not such big deal in times of “big data” to store the original, unprocessed raw data as well. *Molecules* has already been supporting for several years that authors permanently deposit their data in specialized external repositories for future use. But this is not mandatory at present, and it is often complicated, so many authors (including myself) have not started doing so regularly. Making raw data a part of the submission would make things much less complicated. To come back to the journal’s history: the initial purpose of *Molecules*/MDPI was to be a repository of chemical compounds (the original meaning of the acronym MDPI was “Molecular Diversity Preservation International” (Contribution 5)). I like the idea that the “D” in this old acronym could also stand for “Data”. Thus, *Molecules* (and maybe other MDPI journals) could become repositories for the data underlying their publications. So, FORD submission and storage directly within the journal is my second future vision. I do not know whether this one is realistic in the near future, but again, I can foresee that its implementation would definitely help in minimizing the misuse of data and thus save a lot of unnecessary effort and resources at later times. It would remove another obstacle hindering the LOS.

Dear readers, I think you will agree with me that OA publishing, apart from providing unlimited access to all its content, must also be fully open, i.e., maximally transparent, at all its stages of editing and publishing as well as all other features. This is not an easy task, but it is not only desirable, it is mandatory to ensure that serious and honest scientific publishing, as the standing pillar of true science as we know it, can continue to exist in the future. I am convinced that *Molecules*, with its 30 years of very successful publishing history, can continue to be a pioneer in this way for the benefit of science. Looking ahead in this way, with all of us continuing to strive for the quality, reputation and success of our journal, is my major vision for *Molecules*’ bright, transparent and open future in the next 30 years and more! To work! But before that, let us celebrate!

Happy 30th anniversary, *Molecules*, and my sincere regards and good wishes to all its past, present and future contributors.

## Figures and Tables

**Scheme 1:**
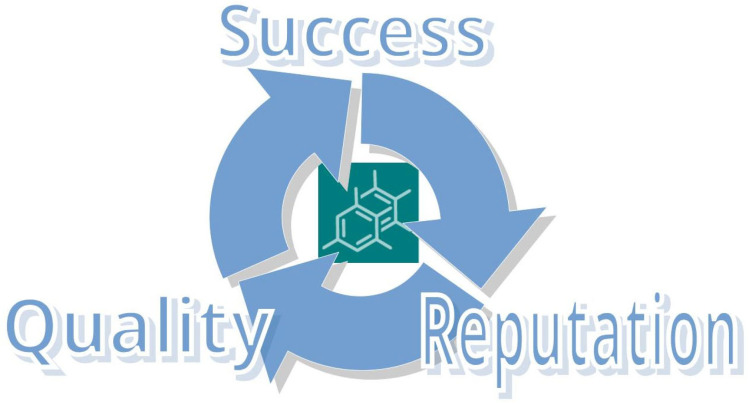
Self-amplifying loop of success (LOS).

